# Disentangling the genetic overlap between ischemic stroke and obesity

**DOI:** 10.1186/s13098-024-01555-x

**Published:** 2024-12-30

**Authors:** Ren Yang, Tangfeng Zhang, Feng Han

**Affiliations:** Department of Neurosurgery, The Affiliated Hospital of Guizhou Medical University, Guiyang, Guizhou Province People’s Republic of China

**Keywords:** Ischemic stroke, Obesity, Genetic correlation, Genetic overlap

## Abstract

**Objective:**

Obesity has been recognized as a risk factor for cerebrovascular diseases, with observational studies suggesting a heightened incidence of stroke. However, the genetic epidemiology field has yet to reach a consensus on the causal relationship and genetic overlap between ischemic stroke (IS) and obesity.

**Methods:**

We utilized linkage disequilibrium score regression, high-definition likelihood, and local analysis of variant associations to assess the genetic correlation between body mass index (BMI) and IS. Bidirectional Mendelian randomization was employed to infer causality. We identified shared risk single nucleotide polymorphisms (SNPs) through cross-trait meta-analyses and estimated heritability using summary statistics. Summary-data-based Mendelian randomization (SMR) was applied to explore potential functional genes.

**Results:**

Our analysis revealed a significant positive genetic correlation between BMI and IS, supporting a causal link from BMI to IS. Cross-trait analysis yielded 9 and 16 shared risk SNPs for IS and small vessel stroke (SVS), respectively. We observed a notable enrichment of SNP heritability for IS and BMI in brain tissues, suggesting tissue-specific influences. The genes shared between the traits were predominantly involved in brain development, synaptic electrical activity, and immunoregulation. Notably, our SMR analysis identified the risk genes CHAF1A, CEP192, ULK4, CYP2D6, AS3MT, and WARS2 across the majority of the 14 enriched tissues shared by both traits.

**Conclusion:**

Our study uncovered a significant genetic correlation and identified shared risk SNPs between BMI and IS. The identification of CHAF1A, CEP192, ULK4, CYP2D6, AS3MT, and WARS2 as potential functional genes common to both obesity and IS enriched our understanding of their genetic interplay, potentially advanced our grasp of their pathogenesis and therapeutic targets.

**Supplementary Information:**

The online version contains supplementary material available at 10.1186/s13098-024-01555-x.

## Introduction

Cerebrovascular diseases, characterized by disruptions in cerebral blood flow and the ensuing neurological impairments, represented a substantial threat to global health. They ranked as primary contributors to mortality and disability across the globe [1]. Among these conditions, stroke, and ischemic stroke (IS) in particular, stands as the second leading cause of death and the third leading cause of disability among adults worldwide [2]. Concurrently, obesity has become a critical public health and economic issue [3]. Observational studies have shown that individuals with a higher body mass index (BMI) have approximately double the risk of experiencing a stroke compared to those of normal weight [4]. Further, mendelian randomization (MR) studies supported the association between elevated BMI and an increased risk of stroke [5], highlighting a bidirectional comorbidity between IS and obesity. Furthermore, previous studies suggested obesity deteriorates the functional outcome after ischemic stroke. But there are researches claiming that obesity is associated with lower mortality, recurrence, and readmission rates, which is known as the obesity paradox [6]. Despite these findings, there remains a need for updated data to elucidate the causal link between obesity and IS, as the shared genetic basis underlying these conditions is not yet fully understood.

By deciphering the complex relationship between obesity and IS, we can pinpoint potential intervention targets and forge more effective prevention and treatment strategies for IS. Proposed mechanisms suggested that obesity may lead to vascular dysfunction and disrupt autonomic regulation of blood pressure, thereby elevating IS risk [7]. Nonetheless, fully comprehending the intricate processes that was correlated obesity and ischemic stroke remained an arduous endeavor.

**Fig. 1:**
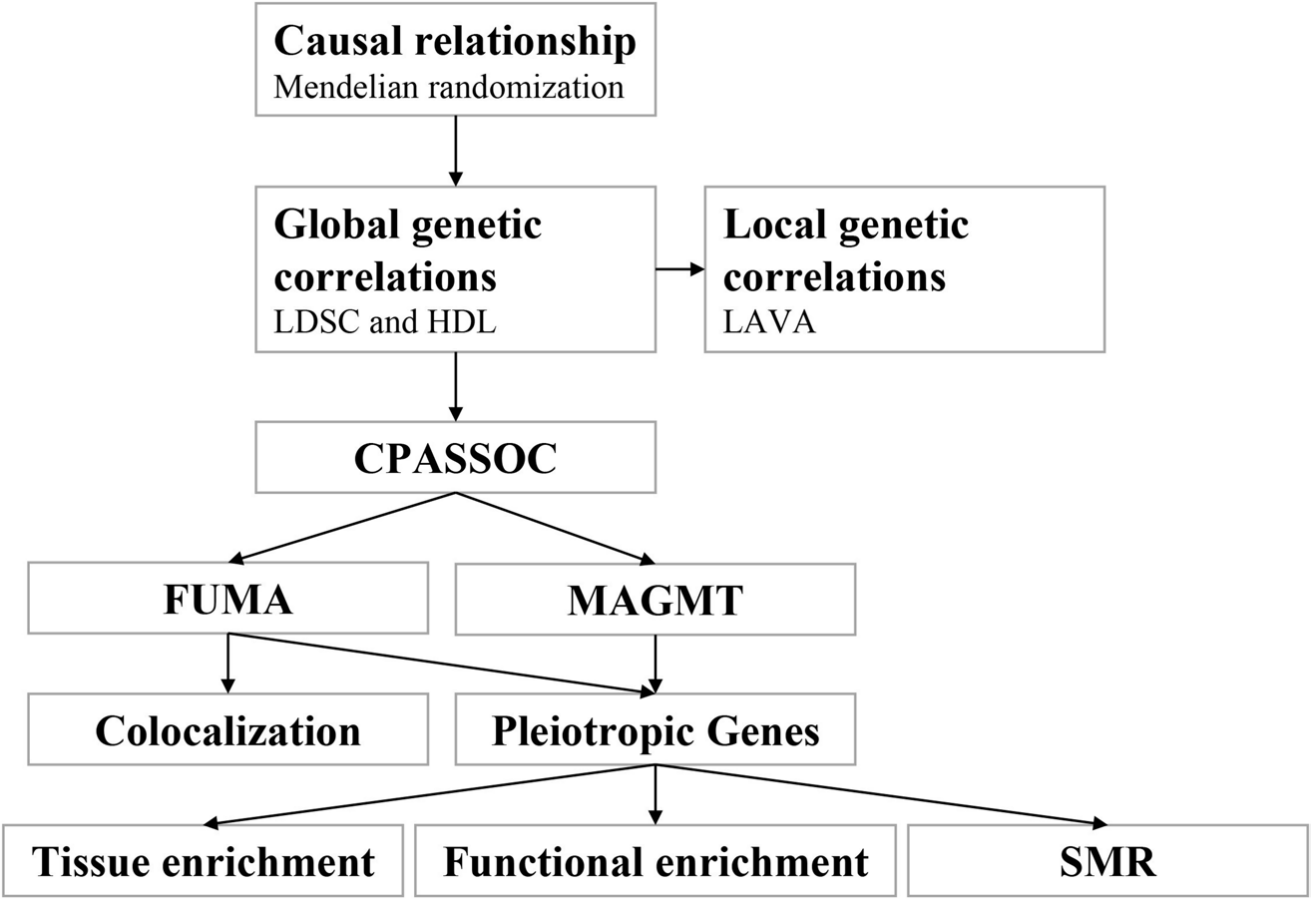
Flow diagram of shared genetic architecture between IS and BMI. IS Ischemic stroke; BMI body mass index; LDSC linkage disequilibrium score regression; HDL high-definition likelihood; LAVA Local analysis of variant association; CPASSOC cross phenotype association; FUMA Functional Mapping and Annotation; MAGMT Multi-marker Analysis of GenoMic Annotation; SMR Summary-data-based Mendelian randomization

In this study, we leveraged summary statistics from a large-scale genome-wide association study (GWAS) to explore the genetic correlation, causal relationship, and shared risk loci with potential functional implications between IS and obesity (Fig. 1). Our findings aimed to deepen the understanding of the comorbidity between these two conditions. We began by examining the genetic correlation to determine the extent to which the same genetic variants influence both IS and obesity. Subsequently, a cross-trait meta-analysis was conducted for a more refined genetic correlation assessment. Furthermore, MR analysis was employed to discern the directionality and potential causality between obesity and IS. We also utilized the Genotype-Tissue Expression (GTEx) dataset to unveil tissue-level SNP heritability enrichment associated with IS and obesity.

## Methods

### Data resources

#### GWAS datasets

The Genetic Investigation of Anthropometric Traits (GIANT) consortium conducted a GWAS meta-analysis involving approximately 0.7 million participants. The analysis included 2.4 million SNPs from the HapMap 2 database. The effect estimates for SNPs associated with BMI were derived using the participants' weight and height information [8]. For the study on IS and its subtypes, the genetic association data was obtained from a previously published GWAS conducted by the MEGASTROKE project [9]. In total, the analysis included 34,217 cases of IS and 406,111 controls. To categorize the IS cases into subtypes, the Trial of Org 10,172 in Acute Stroke Treatment criteria were utilized. Among the IS cases included in the analysis, there were 7193 cases classified as cardioembolic stroke (CES), 4373 cases classified as large vascular stroke (LAS), and 5386 cases classified as small vessel stroke (SVS) [10].

#### Transcriptome data

In our study, we utilized gene expression data obtained from the GTEx project, which is a publicly available resource providing gene expression information across 53 non-diseased human primary tissues [11].We downloaded the GTEx v6p dataset and specifically selected the GTEx V8 expression quantitative trait locus (eQTL) summary data, applying a significance threshold of P < 1 ×10^−5^ [12] for the downstream analysis, we utilized cis-eQTL summary statistics for whole blood, obtained from eQTLGen, which is a meta-analysis involving 14,115 individuals.

### Statistical analyses

#### General genetic correlation analysis

Linkage disequilibrium score regression (LDSC) was used to investigate the genetic overlap between IS and BMI [13]. For the estimation of genetic correlations (rg) between IS and BMI, we used bivariate LDSC with a constrained intercept due to population overlap between the GWAS of IS and BMI. The χ2 statistic was replaced by z-scores, and the genetic covariance was estimated by regressing both z-scores on LD scores[14]. Additionally, we conducted high-definition likelihood (HDL) analysis to estimate genetic correlations between BMI, IS, and SVS. HDL calculations for genetic correlations were based on summary data from GWAS, which was similar to LDSC. However, HDL has been suggested to reduce estimation variance for genetic correlations, making it more effective in identifying potential connections between complex human traits [15].

#### Local genetic correlation analysis

Local analysis of variant association (LAVA) was used for establishing local rg analysis. Traditional global approaches, which only considered the average rg across the genome, might not be able to detect situations where the shared information is limited to certain regions or exhibits contrasting directions at various loci. Thus, in order to gain deeper insights into intricate and dependent genetic associations, we performed multivariate genetic association analysis on all 2495 genomic loci in the entire genome, using pairwise local rg tests.

#### Cross-trait GWAS meta-analysis

To identify the risk SNPs associated with the joint phenotypes of BMI and IS, we employed cross phenotype association (CPASSOC) to conduct a cross-trait meta-analysis of GWAS summary statistics [16]. CPASSOC was used to perform pairwise cross-trait meta-analysis while accounting for variations in the heritability levels of the two phenotypes. It conducts a sample size-weighted meta-analysis of GWAS summary data to estimate the cross-trait statistic heterogeneity (SHet) and p-value, assuming the presence of heterogeneous effects across traits. The SNPs that exhibited significant associations with both phenotypes (P < 5e−8 in CPASSOC) were considered as the significant SNPs. Subsequently, SNPs that showed the most significant associations with the phenotype within a 1000-kb distance on chromosomes were independently selected. SNPs in linkage disequilibrium (LD r^2^ > 0.01) with these selected SNPs were excluded using PLINK v1.9 [17]. Independent SNPs without linkage disequilibrium (LD r^2^ > 0.01 within 1000-kb windows) that were significant in previous GWAS (including the two GWAS used in this study and previous GWAS on BMI and IS) were defined as novel loci.

#### Colocalization analysis

To investigate the shared genetic variants between IS and BMI, we conducted a colocalization analysis. This analysis helps explain the genetic relationship between these two traits by examining the pleiotropy of SNPs. We utilized the colco.abf function from the Coloc package in R to assess whether SNPs obtained from a cross-trait GWAS meta-analysis colocalize, either through shared SNPs or separate SNPs within the same gene. Coloc employs a Bayesian algorithm to calculate posterior probabilities for five mutually exclusive hypotheses related to the sharing of causal variants within a specific genomic region [18]. A locus was considered colocalized when the posterior probability of hypothesis 4 (PPH4) exceeded 0.70.

### Functional mapping and annotation

Functional Mapping and Annotation **(**FUMA) platform [19] was used to obtain annotation information for SNPs associated with functional categories. FUMA can provide relevant information, especially for non-coding or intergenic regions, as GWAS results frequently fail to directly determine causal variants due to the impact of linkage imbalances. Regarding the information, it can be observed that CADD scores higher than 12.37 signify the possibility of negative effects on protein outcomes. Additionally, the scores obtained from RegulomeDB provide valuable understanding of the regulatory functionality of SNPs.

### Tissue and functional enrichment analysis

To further comprehend the biological implications of the final pleiotropic genes identified from the overlapping genes detected by MAGMA and the annotation result in FUMA, an enrichment analysis was conducted on these genes. The analysis focused on Gene Ontology (GO) [20] biological processes and Kyoto Encyclopedia of Genes and Genomes (KEGG) pathways, using the "clusterProfiler" R package. To determine the tissues that are most linked to the shared genes between BMI and IS, we conducted GTEx tissue enrichment analysis. GTEx (v.8) offers information on SNP mutations associated with gene expression quantitative traits in multiple tissues, encompassing a total of 53 tissue types [12].

### Multi-marker analysis of GenoMic annotation

Gene and gene-set analysis, which have been proposed as alternative approaches to the usual single-SNP analysis carried out in GWAS, may potentially have greater efficacy. MAGMA is a tool that is fast and flexible for analyzing genes and gene sets in GWAS genotype data [21]. MAGMA utilized a multiple regression method for gene analysis to accurately account for LD between markers and identify multi-marker effects. In this study on pleiotropic SNPs identified from CPASSOC, it is performed on FUMA. The gene sets generated by MAGMA were compared with the gene sets corresponding to the loci identified in CPASSOC. After implementing the Bonferroni correction, the final set of pleiotropic genes identified at the gene level consists of the resulting genes.

### Summary-data-based Mendelian randomization

Summary-data-based Mendelian randomization (SMR) was employed to identify potential functional genes associated with IS and BMI. SMR is a technique that integrates summary statistics from GWAS and eQTL studies within the MR framework to investigate the relationship between gene expression and a specific phenotype [22]. To assess the presence of linkage in the observed associations, the heterogeneity in dependent instruments (HEIDI) test was performed using genome-wide significant SNPs as instrumental variables. SMR employed the HEIDI-outlier test to distinguish between causality or pleiotropy and linkage. These functional genes passed the Benjamini-Hochberg false discovery rate (FDR) test and the HEIDI outlier test (p > 0.05, N > 10 SNPs).

### Mendelian randomization

MR used SNPs with significant correlations as instrumental variables (IVs) for investigating causal relationships between exposure and outcome. In our study, the primary method used was the inverse-variance weighted (IVW) approach. Using a random effects inverse-variance approach, this method combined the Wald ratio estimate of each SNP, obtained by dividing the SNP outcome estimate by the SNP-exposure estimate, using weights based on the standard error of each ratio. The IVW method calculates average causal effect estimates for both traits. We used the MR-Pleiotropy Residual Sum and Outlier (MR-PRESSO) and MR-Egger regression methods to identify and address horizontal pleiotropy. We analyzed the relationship between the genetic liability of BMI and IS by conducting a reverse-direction MR analysis.

## Results

### Evidence for causality between BMI and IS

**Fig. 2:**
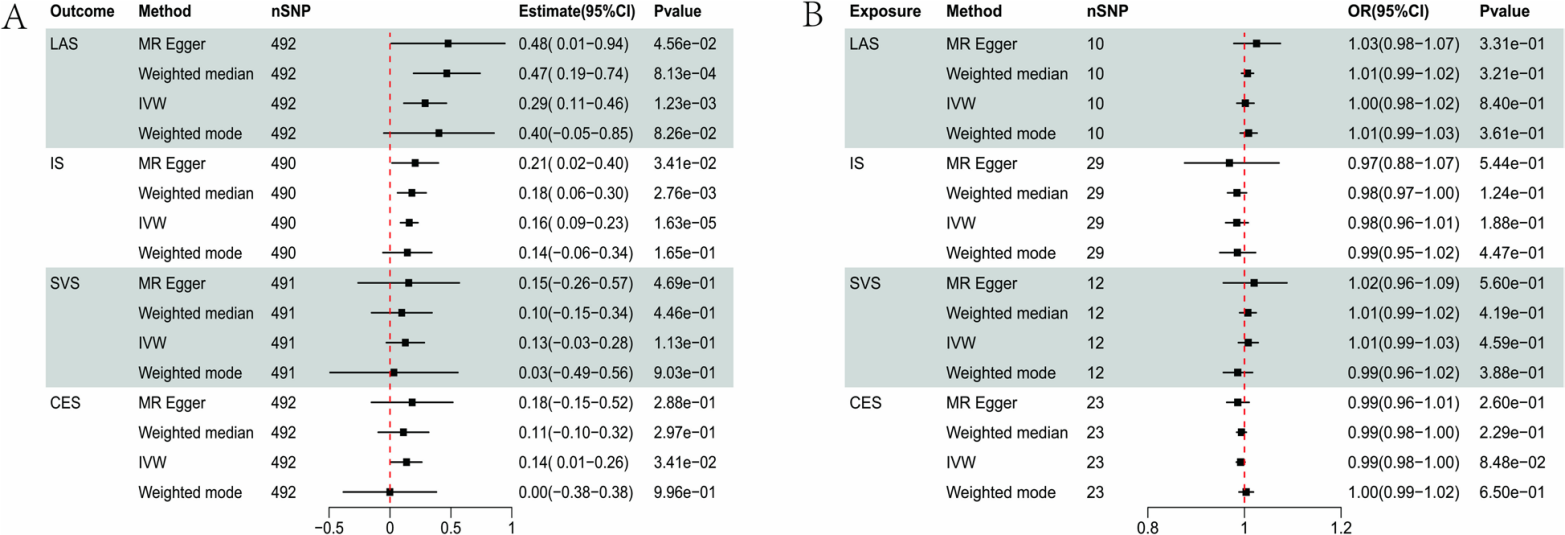
Bi-directional Mendelian Randomization (MR) analyses between IS and BMI. **A** Causal effect of BMI on IS; **B** Causal effect of IS on BMI

We performed a bidirectional MR analysis using the identified loci that demonstrated a significant association with the single phenotype GWAS of IS or BMI as IVs. All SNPs used in the MR analysis exhibited a high instrument strength, with an F-statistic greater than 10. We found evidence to support the causality of BMI on IS in four methods (MR Egger β = 0.21, se = 0.097, p = 0.034; weighted median β = 0.18, se = 0.06, p = 0.0027; IVW β = 0.16, se = 0.037, p = 1.63E−05), but those results with significant heterogeneity (IVW Q = 658.67, p = 4.29E−07). Egger intercept was not different from 0, which suggested that the IVs were not pleiotropic. The leave-one-out analysis showed that no SNP was driving the effect. We also found that BMI may increase the risk of LVS (MR Egger β = 0.477, se = 0.24, p = 0.046; weighted median β = 0.47, se = 0.14, p = 0.0008; IVW β = 0.29, se = 0.09, p = 0.001, Fig. 2) but with significant heterogeneity (IVW Q = 641.24, p =  5.45E−06, Figs. S1 and S2). In other words, the causality of IS and SVS with BMI was not stable. However, we did not observe the casual effect of BMI on SVS and CES. Furthermore, IS and its subtypes did not affect BMI supported by all methods in the reverse analyses (Fig. 2).

### Genetic correlations

LDSC was employed to calculate the liability-scale SNP heritability for BMI and IS and its subtypes. The liability-scale SNP heritability for BMI, IS, SVS, LVS, and CES were 21.2, 0.46, 44.46, 0.76, and 0.87%, respectively. We found that BMI was significantly genetic associated with IS (rg = 0.187, p<0.001) and SVS (rg = 0.281, p<0.001), but no genetic correlation observed between BMI and LVS, and CES, respectively (Table 1). Furthermore, HDL revealed that a significant genetic correlation between BMI and IS (rg = 0.243, p<0.001), and SVS (rg = 0.421, p = 0.0164), respectively.

**Table 1:** The global genetic correlation between BMI and IS

Stroke	r_g_ (SE)	*P*-value	Genetic covariance (SE)	BMI Lambda GC	BMI intercept	IS	IS
						Lambda GC	Intercept
IS	0.1868 (0.0316)	3.41E−09	0.0168 (0.0037)	2.9072	0.9855 (0.0331)	1.0466	0.953 (0.009)
LAS	0.1197 (0.1162)	0.3028	0.0048 (0.0038)	2.7872	1.0222 (0.0295)	1.0165	1.0227 (0.0071)
SAS	0.2809 (0.0609)	3.96E−06	0.0861 (0.0161)	2.7872	1.0257 (0.0287)	1.0225	0.9852 (0.0063)
CES	0.1087 (0.0562)	0.5298	0.0051 (0.0023)	2.7872	1.0233 (0.0261)	1.0213	1.0331 (0.0045)

*IS* Ischemic stroke, *BMI* body mass index, *LAS* large vascular stroke, *SAS* small vessel stroke, *CES* cardioembolic stroke

### Local genetic correlations

The single GWAS was partitioned into 2495 loci to conduct the LAVA analysis, which aimed to explore the genetic correlation between IS and BMI. The threshold equals p = 0.05/2495, which is equivalent to 2.00E−5. Strong local correlations were found in two loci (chr22 27192924–27952441, p = 4.37E−06; chr18 6756497–7862479, p = 1.45E−05) for SVS with BMI (Table S1). However, we did not identify the local genetic association between BMI and IS because of a lower liability-scale SNP heritability of IS.

### Identification of shared SNPs by cross trait meta-analysis

**Fig. 3:**
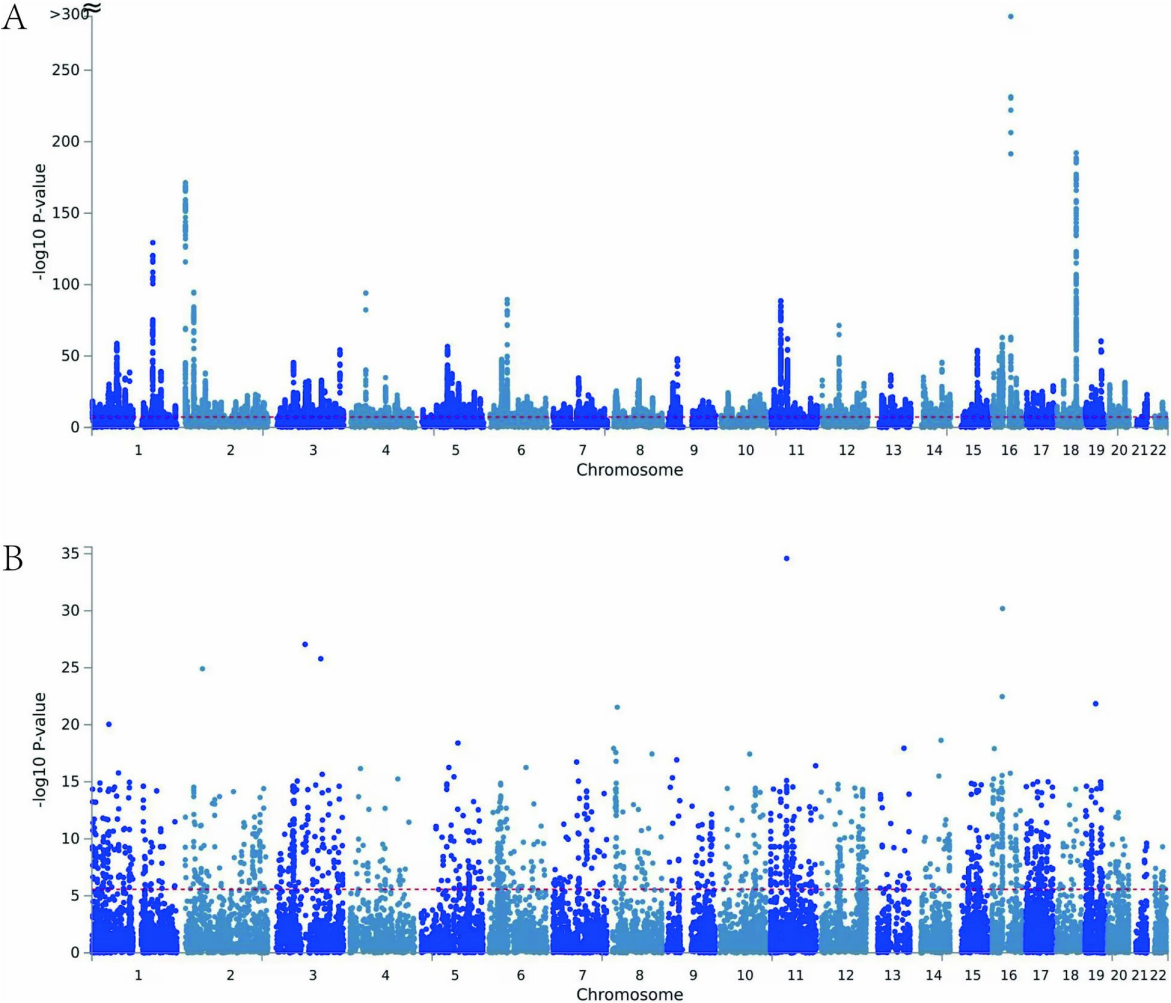
Manhattan plot showed the pleiotropy SNPs and genes revealed by CPASSOC (**A**) and MAGMA (**B**)

We then employed the CPASSOC and set a threshold of p < 5E−8 for meta-analysis to screen a pleiotropic SNP. A total of 36334 overlap SNPs were identified among IS and BMI (Table S2 and Fig. 3), 41441 were observed for SVS and BMI (Table S3). The SNP with the most significant statistic between BMI and IS, and BMI and SVS were rs7206790 (p = 1.165E−312) and rs7235626 (p = 3.26e−99). There were 9 novel SNPs shared between IS and BMI (p_BMI_ and p_IS_<5E−6 and p_CPASSOC_ <5E−8, Table S4) and 16 novel SNPs for SVS and BMI after excluding SNPs (Table S5) that were significant in the single-trait GWAS of IS or SVS, or BMI, or were in LD (LD r^2^ ≥ 0.02) with any of previously reported significant SNPs. We then employed FUMA platform to calculate the annotation information of the shared SNPs between two traits, respectively. A total of 784 genomic locus with 4529 independent significant SNPs (Table S6) and 1584 lead SNPs (Table S6) were identified between IS and BMI. There were 2797 genes were mapped according to the results of CPASSOC for IS and BMI (Table S6). However, there were 337 genomic locus with 1457 independent significant SNPs and 505 lead SNPs were obtained for SVS and BMI (Table S7). A total of 1337 genes were mapped based on the results of CPASSOC (Table S7).

Finally, we also used MAGMA to map the common SNPs between IS and BMI. There were 18,483 and 18,689 genes were identified for BMI and IS, BMI and SVS, respectively (Table S8 and S9, Fig. 3). The intersect genes with Bonferroni correction mapped via MAGMT and FUMA were used for tissue and functional enrichment analysis (Table S10 and S11).

### Colocalization analysis

We conducted colocalization analysis to confirm if the genomic region has pleiotropic effects on the loci annotated on FUMA. The results indicated that 4 loci (including 570, 42, 75 and 263) were associated with IS with a PPH4 value exceeding 70%. The significant SNPs included rs11066301, rs11066028, rs233721, rs2891403, rs16864515, rs12759907, rs235509, rs6893539, rs11241696, and rs7711753, which were used to map 17 genes including SH2B, ATXN2, BRAP, ACAD10, RP11-162P23.2, ALDH2, MAPKAPK5, TMEM116, ERP29, NAA25, TRAFD1, HECTD4, RPL6, PTPN11, RPH3A, PRRC2C and CEP120 (Table S12). However, no genomic region has pleiotropic effects on the loci for SVS (Table S13).

### Tissue and functional level SNP heritability enrichment

**Fig. 4:**
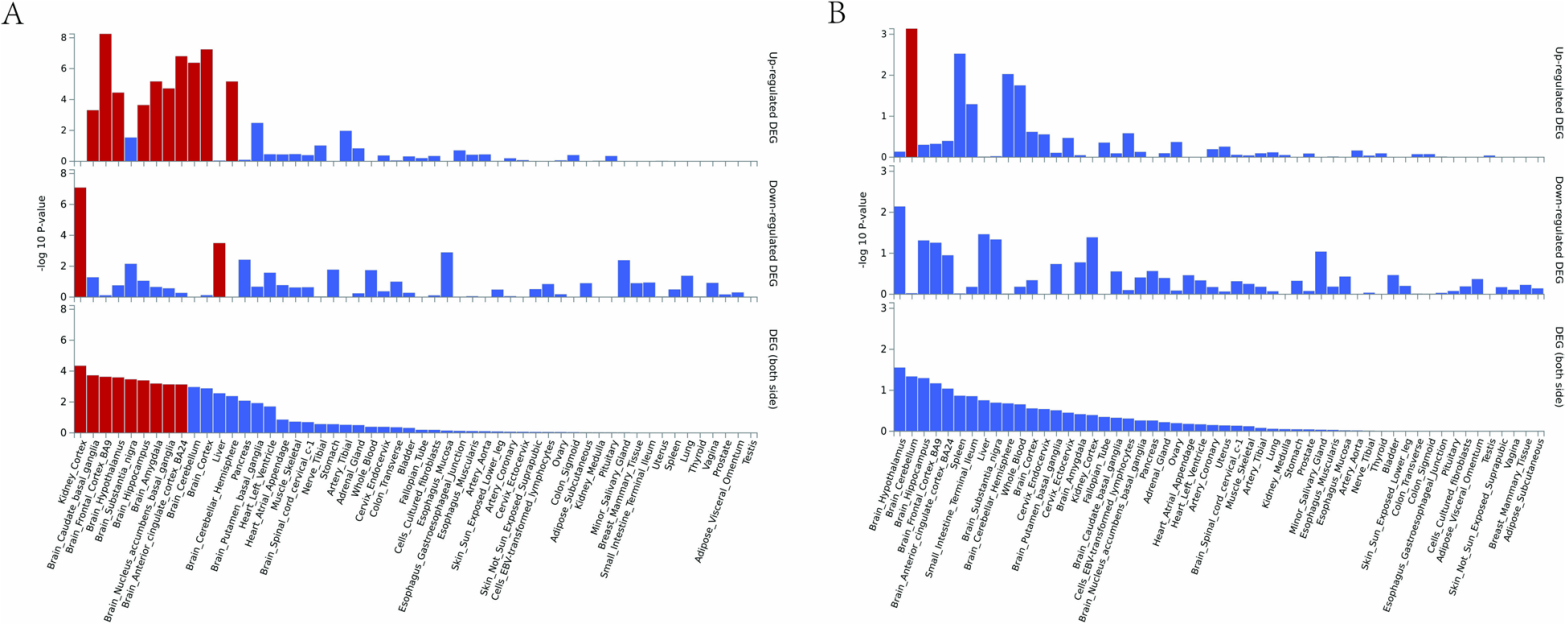
MAGMA-based heritability enrichment estimates in tissues for BMI and IS. **A** IS, **B** SVS

The intersect genes with Bonferroni correction mapped via MAGMA and FUMA were used for tissue and functional enrichment analysis. MAGMA was employed to explore the tissue-level SNP heritability enrichment for IS and BMI, using GTEx with different tissues. We discovered that the brain tissues exhibited shared significant SNP-heritability enrichment for both IS and BMI (Table S14 and Fig. 4). In details, we found that SNPs associated with BMI and IS were enriched in 18 different brain regions, leading by frontal cortex, anterior cingulate cortex, cerebellum, and amygdala. However, the shared SNPs among BMI and SVS were only enriched in cerebellum (Table S14 and Fig. 4).

**Fig. 5:**
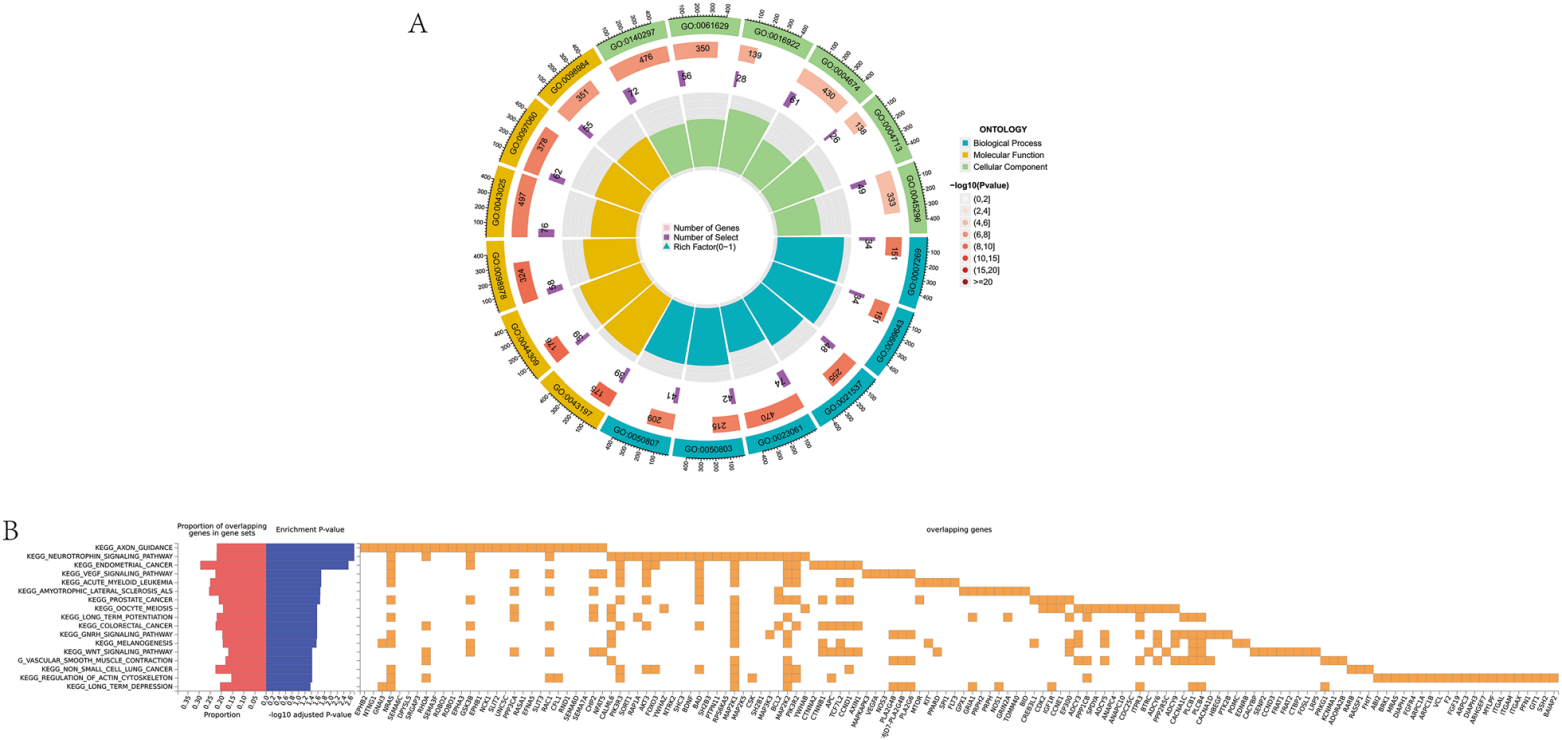
Functional enrichment for pleiotropy genes between IS and BMI. **A** GO analysis, **B** KEEG analysis

Furthermore, we found that the common SNPs between BMI and IS were mainly enriched in the biological processes such as brain development and synaptic electrical activity revealed by GO analysis (Fig. 5 and Table S15). Results of KEEG suggested that those SNPs were enriched in biological pathway such as axon guidance, neurotrophin signaling pathway, and neurosynaptic potential regulation (Fig. 5 and Table S16). Moreover, the genes annotated by the shared SNPs between BMI and SVS were primarily enriched in immunoregulation such as T cell differentiation and mononuclear cell differentiation revealed by GO analysis (Table S15). Those genes were enriched in the signaling pathway involved with immunoregulation demonstrated by KEEG analysis (Table S16).

### Identification of shared functional genes for IS and BMI

In order to infer causality and identify the putative functional genes for BMI and IS, we analyzed GWAS summary data from GTEx using SMR based on the overlapped genes from results of FUMA and MAGMA. A total of 6, 2, 15, 4, 9, 25, 33, 24, 18, 11, 13, 15, 13 and 6 shared genes associated with BMI and IS in aorta artery, coronary artery, caudate basal ganglia, amygdala, anterior cingulate cortex BA24, cerebellar hemisphere, cerebellum, cortex, frontal cortex BA9, hippocampus, hypothalamus, nucleus accumbens basal ganglia, putamen basal ganglia, and substantia nigra, respectively. Among those genes, CHAF1A, CEP192, ULK4, and WARS2 were found in most of the 14 shared enriched tissues. Regarding the common genes between SVS and BMI, we identified 1(WNT3), 1(WNT3), 2(CYP2D6 and FUT2), 3(AS3MT, CYP2D6 and LRRFIP2), 2(AS3MT and CYP2D6), 3(ADAMTSL3, CISD2, and TYW5), 5(ADAMTSL3, CISD2, FAM212A, MANBA, and RNF123), 5(ADAMTSL3, AS3MT, CYP2D6, LRRFIP2 and NAGA), 2(AS3MT and CYP2D6), 2(AS3MT and CYP2D6), 4(CISD2, CYP2D6, MAMSTR and NAGA), 5(AS3MT, CYP2D6, FUT2, LRRFIP2 and MAMSTR), 4(CYP2D6, FUT2, LRRFIP2, and MAMSTR), and 1(CYP2D6) shared genes in aorta artery, coronary artery, caudate basal ganglia, amygdala, anterior cingulate cortex BA24, cerebellar hemisphere, cerebellum, cortex, frontal cortex BA9, hippocampus, hypothalamus, nucleus accumbens basal ganglia, putamen basal ganglia, and substantia nigra, respectively. However, only CYP2D6 and AS3MT were found in most of the 14 shared enriched tissues (Table S17).

## Discussion

Based on our current understanding, this study represented the first comprehensive genome-wide analysis that systematically investigated the shared genetic structures connecting IS and BMI. Our research employed large-scale GWAS datasets and tissue-specific expression data to demonstrate the genetic interplay between IS and BMI. Notably, we have observed a significant association between IS and BMI within specific genomic regions and functional elements. Moreover, we have identified several genes that exhibit pleiotropic effects on both IS and BMI, suggesting their involvement in the development of both phenotypes. Importantly, our bi-directional MR analysis has revealed a causal effect of BMI on IS, suggesting that higher BMI may contribute to an increased risk of developing IS. However, we did not find evidence of a causal effect of IS on BMI. Lastly, our focus has been on exploring the shared genetic characteristics of IS and BMI specifically in brain tissue, leading us to identify several genes that could potentially play a functional role in both phenotypes.

Obesity, which was identified as a risk factor for cerebrovascular disease, have been suggested to be linked to a higher occurrence of stroke [23, 24]. For example, data collected from a total of 97 prospective cohort studies revealed a correlation between being overweight or obese and a higher likelihood of experiencing a stroke [25]. However, the causal association between IS and BMI remained controversial in genetic epidemiology [26, 27]. Our analysis revealed that elevated BMI may play a role in the development of IS and LVS, but not SVS and CES. These findings aligned with previous observational studies and meta-analyses that have frequently reported the co-occurrence of IS and obesity in clinical settings. By utilizing robust GWAS data and conducting comprehensive analyses, our study provided further support for the association between high BMI and the risk of IS and LVS. These results contributed to the growing body of evidence highlighting the relationship between obesity and stroke [24, 28].

Currently, a comprehensive analysis of the genetic association between IS and BMI has not yet been conducted. We discovered a substantial genetic relationship between IS and BMI, thus reinforced the theory that genetic factors have a crucial impact on the comorbidity of IS and obesity. From CPASSOC analysis, we identified 9 suggestively significant SNPs for BMI and IS, and 16 novel SNPs for BMI and SVS. As for SNPs for IS and BMI, rs11065987 and rs3184504 were reported to be associated with hypertension [29, 30], rs11066301 and rs653178 were related to coronary artery disease [31, 32], rs11066320 was associated with LDL cholesterol level [33]. Those phenotypes were involved with stroke and obesity, which also supported the hypothesis that genetic factors affect comorbidity of obesity and IS.

In our study, we used the GTEx datasets to investigate the functional enrichment of gene expression across multiple tissues. Our analysis revealed significant SNP heritability enrichment for BMI and IS in eight tissues, with a prominent enrichment observed in the brain. This indicated that genetic variants associated with BMI and IS were more likely to influence gene expression in brain tissues. Additionally, our findings supported previous suggestions that loci associated with obesity are enriched in both blood vessels and brain tissues. This aligns with existing research highlighting the involvement of these tissues in the genetic regulation of obesity-related traits [34], which implied that obesity may lead to the occurrence of IS by disrupting blood vessels and altering the microstructure of brain tissue. The shared genes for FUMA and MAGMA were then used to perform GO and KEEG analysis. We found that genes annotated by the shared SNPs were mainly enriched in brain development, synaptic electrical activity, and immunoregulation. All these biological processes were associated with the comorbidity of obesity and IS [35], which may contribute to understanding the underlying causes of obesity and IS.

Furthermore, we examined whether shared risk genes can mediate the association between BMI and IS by used blood and tissue eQTL data. Based on SMR and HEIDI, our findings indicate that CHAF1A, CEP192, ULK4, CYP2D6, AS3MT, and WARS2 were present in the majority of the 14 enriched tissues shared between the two traits, suggesting that these genes may serve as a potential connection between the two traits. ULK4 and CYP2D have been reported to be associated with blood pressure and hypertension [36, 37]. As a tryptophanyl-tRNA synthetase, WARS2 may function as a crucial factor in angiogenesis within the heart and other tissues, potentially serving as a coordinator of pro-angiogenic signals, guiding cellular movement and multiplication to facilitate the migration and growth of vascular endothelial cells [38], which indicated that WARS2 may mediate the association between BMI and IS by destructing the cerebral vascular microstructure. CHAF1A, CEP192, and AS3MT were reported to be associated with tumor progression involved with several tumors, such as hepatocellular carcinoma [39] and lung cancer [40] Further explorations are needed to investigate the biological mechanisms of these 6 shared genes on IS and obesity. Recently, some studies have demonstrated a different pattern of circulating non coding miRNA-195-5p and −451a in acute IS and stroke including transient ischemic attack and intracerebral hemorrhage [41–43]. However, whether there is an interplay between these non-coding miRNAs and the genes uncovered by this paper, or those involved in the complex pathogenetic chain linking IS to obesity, remains to be verified through further studies.

Although several investigations have revealed the causal association between IS and BMI in genetic epidemiology [26, 27], evidence on their common genetic structure was scarce. Globally, the continued rise in obesity prevalence posed a significant danger to public health, but there were currently no effective measures to prevent the occurrence of IS. Hence, our study emphasized the significance of identifying and preventing IS at an early stage, especially in individuals who are overweight or obese. Exploring new therapeutics for the two diseases had promising potential for developing modalities that target both diseases based on their shared genetic architecture including CHAF1A, CEP192, ULK4, CYP2D6, AS3MT, and WARS2.

Our study has several limitations that should be taken into consideration. Firstly, the generalizability of our results to other ethnic groups may be limited since the data sources primarily consisted of individuals of European descent. Therefore, it is crucial to conduct similar analyses in diverse populations to validate our findings and develop targeted prevention strategies. Secondly, we were unable to observe a local genetic relationship between IS and BMI due to the lower liability-scale SNP heritability of IS. Consequently, further investigations are needed to explore the genetic connections between IS and BMI in more detail. Lastly, we did not include analyses on the sex chromosomes in our study. This omission was due to the statistical methods used, which were not applicable to sex chromosome analyses.

## Conclusion

In summary, our study revealed a significant genetic correlation and identified shared risk SNPs between BMI and IS. Several potential functional genes, including CHAF1A, CEP192, ULK4, CYP2D6, AS3MT, and WARS2, were identified as common genetic factors linking obesity and IS. These findings provide valuable insights into the genetic basis underlying the relationship between obesity and IS, contributing to a better understanding of their development and potential therapeutic targets.

## Supplementary Information

Additional file 1.

Additional file 2.

Additional file 3.

Additional file 4.

Additional file 5.

Additional file 6.

Additional file 7.

Additional file 8.

Additional file 9.

Additional file 10.

Additional file 11.

Additional file 12.

Additional file 13.

Additional file 14.

Additional file 15.

Additional file 16.

Additional file 17.

Additional file 18.

Additional file 19.

## Data Availability

GWAS summary statistics for BMI are available by application from: http://portals.broadinstitute.org/collaboration/giant/index.php/GIANT_consortium_data_files. GWAS statistics of stroke were obtained from MEGASTROKE consortium (https:// www. megas troke. org/). The eQTL summary data for eQTLGen and GTEx are available from: https://www.eqtlgen.org/cis-eqtls.html; http://yanglab.westlake.edu.cn/software/smr/#eQTLsummarydata.

## Abbreviations

IS: Ischemic stroke
BMI: Body mass index
SNPs: Single nucleotide polymorphisms
SMR: Summary-data-based Mendelian randomization
SVS: Small vessel stroke
MR: Mendelian randomization
GWAS: Genome-wide association study
GTEx: Genotype-Tissue Expression
GIANT: Genetic Investigation of Anthropometric Traits
LAS: Large vascular stroke
eQTL: Expression quantitative trait locus
LDSC: Linkage disequilibrium score regression
HDL: High-definition likelihood
LAVA: Local analysis of variant association
CPASSOC: Cross phenotype association
PPH4: Posterior probability of hypothesis 4
FUMA: Functional Mapping and Annotation
GO: Gene Ontology
KEGG: Kyoto Encyclopedia of Genes and Genomes
HEIDI: Heterogeneity in dependent instruments
IVs: Instrumental variables
MR-PRESSO: MR-Pleiotropy Residual Sum and Outlier
