# Supplementary material for: Disentangling the genetic overlap between ischemic stroke and obesity
Source: Diabetol Metab Syndr. 2024 Dec 30;16:314. doi: 10.1186/s13098-024-01555-x (PMC11684051; doi:10.1186/s13098-024-01555-x)
Supplement: Supplementary file 1 — Additional file 1. [file 13098_2024_1555_MOESM1_ESM.docx]

**Supplementary information**

Figure S1 Pleiotropy, heterogeneity and leave one out analysis for the results of effect of BMI on IS.

Figure S2 Pleiotropy, heterogeneity and leave one out analysis for the results of effect of BMI on LVS.

Table S1 The local genetic correlation between SVS and BMI.

Table S2 Identification of SNP with a p <5e-08 by CPASSOC between IS and BMI.

Table S3 Identification of SNP with a p <5e-08 by CPASSOC between SVS and BMI.

Table S4 Genome-wide significant SNPs in CPASSOC method between IS and BMI.

Table S5 Genome-wide significant SNPs in CPASSOC method between SVS and BMI.

Table S6 FUMA annotated genes associated with IS and BMI.

Table S7 FUMA annotated genes associated with SVS and BMI.

Table S8 MAGMA annotated genes associated with IS and BMI.

Table S9 MAGMA annotated genes associated with SVS and BMI.

Table S10 The intersect significant genes among FUMA and MAGMA results for BMI and IS.

Table S11 The intersect significant genes among FUMA and MAGMA results for BMI and SVS.

Table S12 The colocalization analysis for shared SNPs for IS and BMI.

Table S13 The colocalization analysis for shared SNPs for SVS and BMI.

Table S14 MAGMA-based heritability tissues enrichment estimates for BMI and IS.

Table S15 GO analysis for BMI and IS

Table S16 KEEG analysis for BMI and IS.

Table S17 Identification of shared functional genes for IS and BMI by SMR in 14 shared tissues.
