# Supplementary figures and images for: Disentangling the genetic overlap between ischemic stroke and obesity
Source: Diabetol Metab Syndr. 2024 Dec 30;16:314. doi: 10.1186/s13098-024-01555-x (PMC11684051; doi:10.1186/s13098-024-01555-x)

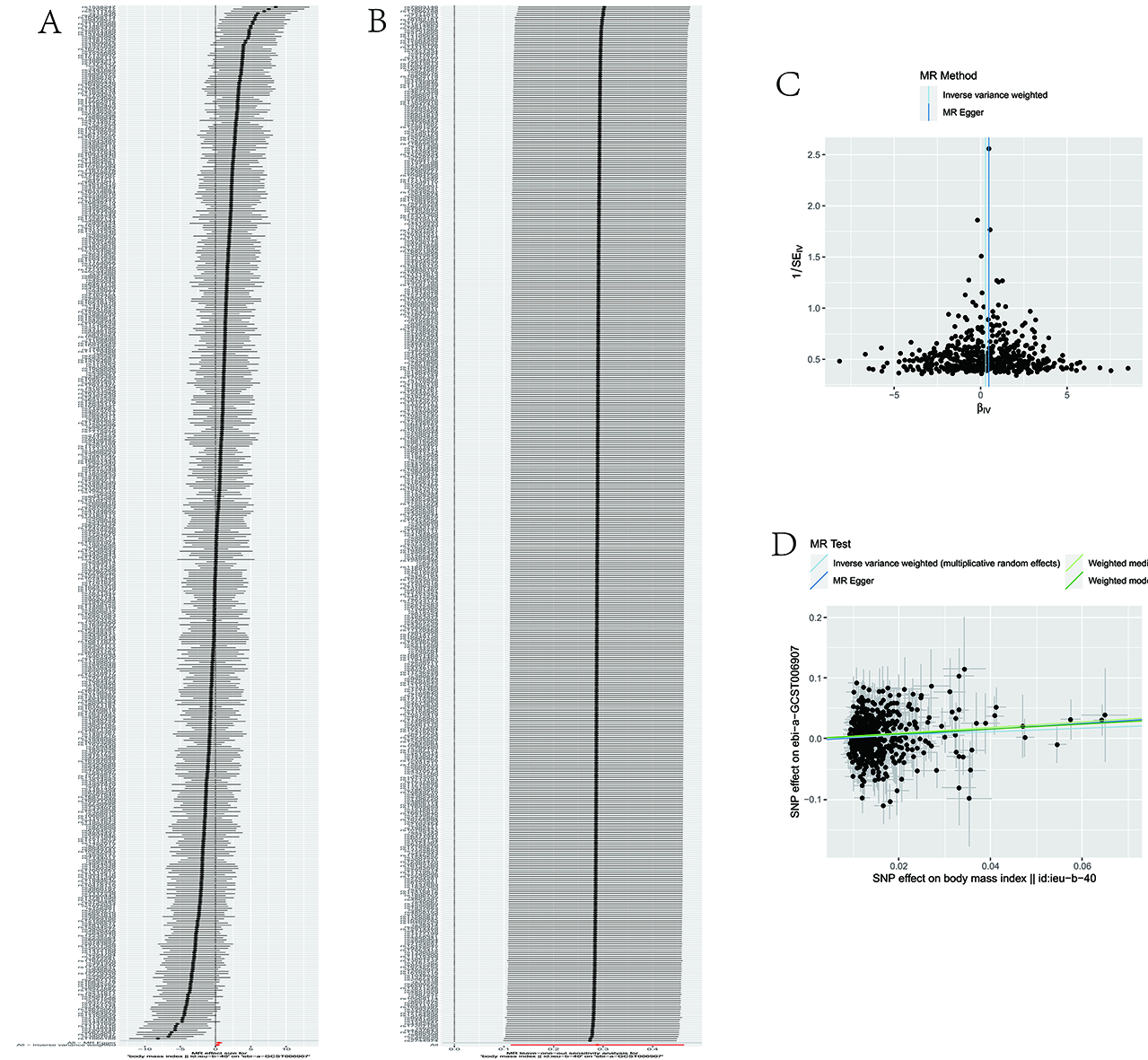

Supplement: Supplementary file 2 — Additional file 2. [file 13098_2024_1555_MOESM2_ESM.tif]

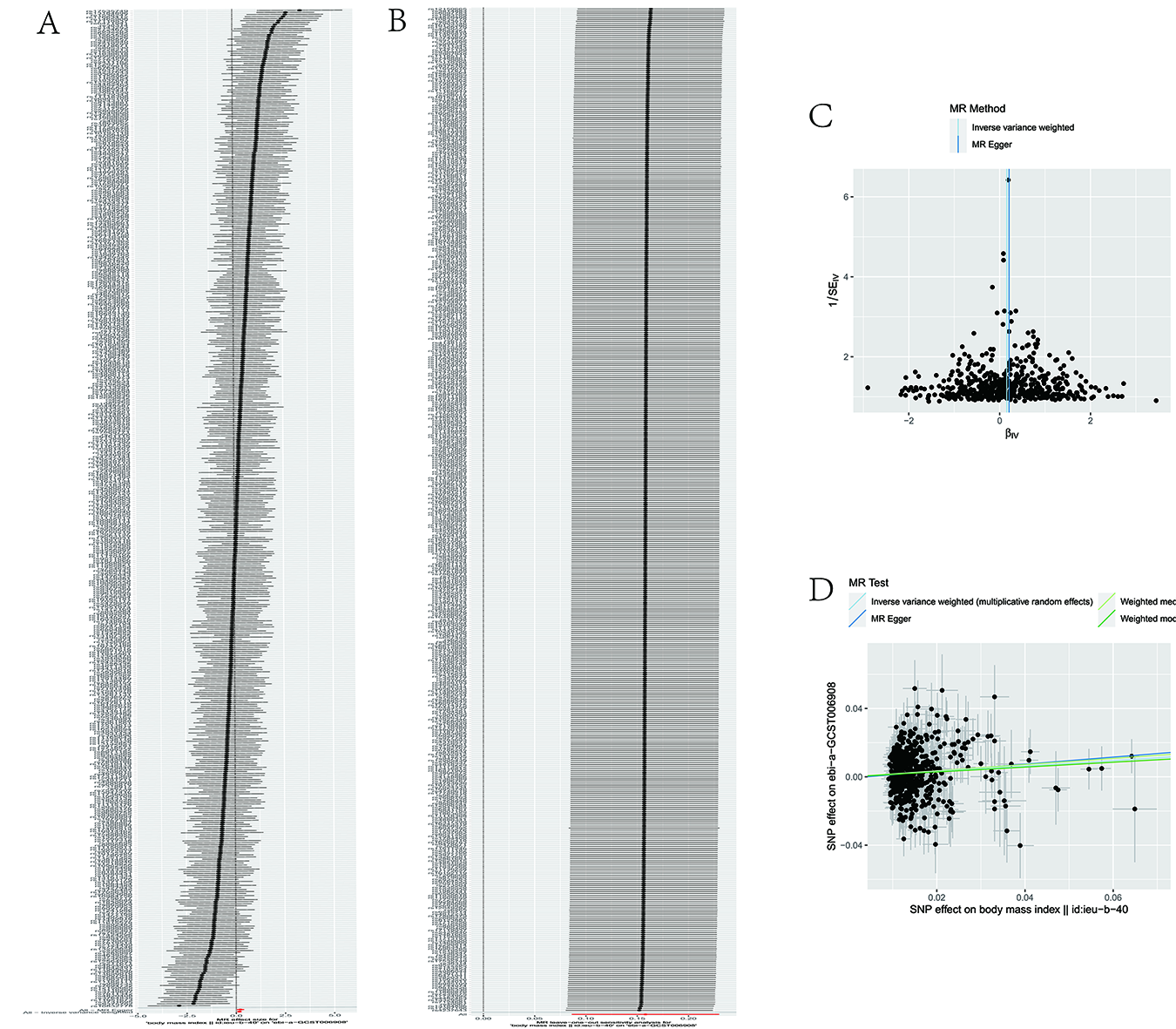

Supplement: Supplementary file 3 — Additional file 3. [file 13098_2024_1555_MOESM3_ESM.tif]
